# Description, Organization, and Individual Postgraduate Perspectives of One Italian School of Anesthesia and Intensive Care

**DOI:** 10.3390/ijerph191912645

**Published:** 2022-10-03

**Authors:** Matteo Villani, Valentina Lob, Anna Del Prete, Emmanuele Guerra, Elisabetta Chili, Elisabetta Bertellini

**Affiliations:** 1 Department of Anesthesia and Intensive Care, Azienda USL Piacenza, 29121 Piacenza, Italy; 2 School of Anesthesia and Intensive Care, University of Modena and Reggio Emilia, 41121 Modena, Italy; 3 Department of Anesthesia and Intensive Care, Azienda USL Carpi, 41012 Modena, Italy; 4 Anesthesia and Intensive Care, Azienda Ospedaliero-Universitaria Modena, 41214 Modena, Italy

**Keywords:** medical training, education, anesthesia and intensive care

## Abstract

Introduction: The study aims to describe the organization of one accredited school of Anesthesia and Intensive Care of University of Modena and Reggio Emilia, Italy. The analysis of the post-graduation period aims to measure the time-to-first job, the perceived challenges, what postgraduate residents choose as first employ, and the overall satisfaction rating of a cohort of residents completing their training until 2017 with the usual and standard training program. Methods: We collected organization and administrative records of the five-year program of the A-IC School of 4 cohorts of residents who joined from 2009 to 2012 and we performed a survey. We also analyzed the differences among school cohorts during the medical training. In the end, it was investigated as a reason to choose hub hospitals or not. Results: The focus of the training activities revolved around the operating room with a mean of 30.41 ± 6.6 (sd), months followed by Intensive care with 17.29 ± 4.49 (sd) months. Although 7.5% of the respondents were not fully satisfied of the school’s program, 89.7% of residents rated their training as adequate. In fact, 97.2% respondents reported they could overcome the professional challenges they faced after graduation. The multiple variables logistic regression showed a correlation among working in hub hospitals and training performed in university hospitals with a *p* value of 0.015. Conclusion: This paper describes the postgraduation period. This point should be examined as an integral part of the accreditation procedure. Knowing the satisfaction rate, perception autonomy, and which type of hospitals are preferred can measure the education training capacity of a postgraduation school.

## 1. Introduction

The continuous and growing demand for specialists in Anesthesia and Intensive Care (A-IC), calls for an analysis focusing on the training programs of A-IC residents [1,2,3]. Today there are 39 schools of A-IC in Italy, with an expanding array of fields of application, such as peri-operative care, Acute and Chronic Pain Therapy, Palliative Care, Hyperbaric Medicine, Emergency Toxicology, in-hospital and out-of-hospital emergencies, and the Coordination of the organ transplant process. In the last ten years the number of residents of A-IC school has increased from the concomitant lack of staff due to a high retirement number of anesthesiologist and resuscitators and inadequate turnover and the pandemic event. Only later accreditation procedure from the Italian Minister of Education and Research (MIUR) it is possible for the university to receive eligibility to be a school of A-IC.

The MIUR classifies A-IC training as a specialized clinical service that develops over the course of five years. Residents are supervised by a tutor in the development of technical skills and competencies, the knowledge acquisition is tracked with an educational credit system [4,5,6]. Unlike other European countries, A-IC in Italy includes the pre-hospital emergency medicine, pain management, and hyperbaric therapy. Future residents are admitted to A-IC schools according to a national ranking. The total number of scholarships available varies each year depending on the estimated need of professionals. Each school is granted a batch of scholarships based on its educational capacity, calculated with an algorithm that considers the number of qualified training posts it can offer across its network. The MIUR asks every school of A-IC to have the chance to attend all fields of anesthesia and intensive care in Italy. Residents usually attend the central core of medical training and education in hub university hospitals and complete the instruction in peripheral spoke hospitals or abroad. Moreover, the MIUR asks every resident to attend several procedures to reach complete autonomy. The accreditation procedure does not consider many factors, including the individual perspectives and satisfaction of the resident’s later postgraduate school, time to first employment, and type of hospital choice. A postgraduation school should prepare all residents to work in all hospitals, from hub hospitals with a high volume of procedures and intensive care units in peripheral and private hospitals.

The accreditation procedure does not consider the postgraduation period. Few data are available about the postgraduation period and individual perceptions about new specialist autonomy and preparedness. Furthermore, it could be a difference between theoretical aspects of accreditation procedure and practical results of medical training.

### Aims of the Study

The principal goal of this study is to assess the educational survey of the A-IC School of Modena and Reggio Emilia, Italy to try to consider these factors: individual perspectives and satisfaction of the resident’s later postgraduate school, time to first employment, and type of hospital choice.

The secondary outcome is firstly checking if cohorts received similar education, then searching if there is any reason to choose hub hospitals or different hospitals, such as peripherals or private hospitals.

## 2. Materials and Methods

We collected organization and administrative records of the five-year program of the A-IC school of 4 cohorts of residents who joined from 2009 to 2012. We collected all data from the official individual training schedule and administrative data. We classified each activity into one of the following categories: Operating Room Anesthesia at University Hospital (ORAUH), Intensive and Emergency Care in University Hospitals (IECUH), activities, and medical training in school network and/or abroad in different countries. The University of Modena and Reggio Emilia (UNIMORE) A-IC school is centered on the two Hub Hospitals of the Province of Modena (Azienda Ospedaliero-Universitaria of Modena, Italy) supported by all spoke hospitals. The ASMN Hospital in Reggio Emilia, Italy, is the third hub hospital that in recent years has joined the training network [7]. The Hyperbaric Medicine Center in Bologna, Italy offers training opportunities in Hyperbaric Medicine, as show in Table 1. An established collaboration network of foreign healthcare facility offers older residents (3rd year and above) a wide variety of high-profile, specialized training opportunities, such as Cardiac Surgery and Pediatric Anesthesia (Table A1 and Table A2). Operating Room Anesthesia includes the perioperative anesthesia management of patients for the following surgical fields: General surgery, Orthopedics Obstetrics and Gynecology, Urology, Otolaryngology, Oral Cranio-Maxillo-surgery, Plastic Surgery, Pediatrics, Vascular surgery, Neurosurgery, Cardiac Surgery, Transplant Surgery, Endoscopy Gastroenterology, Operative Radiology, postoperative pain management, and chronic pain management. Intensive Care and Resuscitation includes the following fields: Post-operative Intensive Care, General Intensive Care, Trauma Intensive Care, Echo-cardio Lab, Radiology Unit, Nephrology Unit, Stroke Unit, and Hyperbaric medicine. Table A3 and Table A4 summarize the standard school programs. Each resident must attend some obligatory disciplines and some optional, but if the resident agrees, part of them can also be attended in the school network and not only in the main university hospital.

The official school records were used to analyze the individual training program for each resident. The time-to-first job and hospital type was obtained from the first documented public evidence of employment. A brief overall satisfaction questionnaire was sent to each resident to confirm employment data, assess the perceived professional challenges, and rate them on a scale from 0–10, where 0 indicated no challenge at all and 10 was the most demanding challenge imaginable. The overall satisfaction section focused on the adequacy of the training towards the requests of the job market: participants were asked to rate their satisfaction on a scale from 0 to 10, where 0 was not satisfied at all and 10 was fully satisfied.

First employment was considered as stable employment with a contract of at least one year or time necessary to start to work or the time needed to start working as a freelance. We also analyzed the eventual difference among school cohorts during the medical training. In the end, the reason to choose hub hospitals or not was investigated. Data were analyzed with Stata^®^ software 16 and GraphPad Prism version 9.0 for Windows, GraphPad Software, San Diego, CA USA, www.graphpad.com (accessed on 23 June 2022)”. Data are expressed as mean plus standard deviation (sd) or percentage. Variance Analysis or a non-parametric Kruskawall test was performed in case of a different variance or normality among school cohorts. In the end, we compared the group of those who work in Hub Hospitals and those who do not. Multivariable logistic regression was performed to understand the reason to work in hub hospitals, the dependent variable was considered working in hub hospitals, and independent variables (ORAUH IECUH and time to first employment). Time spent in university hospitals during training was investigated as was a possible correlation between the future choice of hub hospitals. Multicollinearity was checked and received the operating curve of the logistic model. Margin estimation was performed to plot the probability of working in hub hospitals. Confidence intervals (CIs) were calculated at 95%. All differences with a *p*-value (*p*) ≤ 0.05 were considered statistically significant. 

## 3. Results

### 3.1. First Endpoint

The first job was in a hub hospital in 46.14% of the alumni, in a spoke hospital in 34.15%, and 19.51% in a private hospital, while no difference were present in school cohorts (Table 1). 

**Figure 1:**
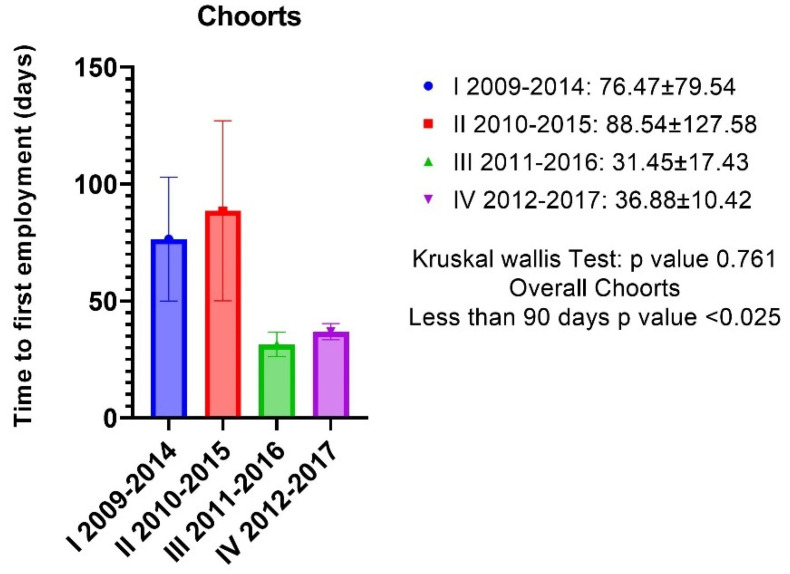
There were no differences in terms of time-to-first-job based on the cohorts. The mean time-to-first-job was under 90 days and specifically 60.28 ±79.73 (sd), CI (34.43–86.12) with a *p*-value of 0.025.

The response rate to the overall satisfaction questionnaire was 87.25% (42/48 subjects); 3 people refused to participate and 3 did not submit their answers. In total, 92.5% of subjects were satisfied with the school program, 7.5% were not completely satisfied; some 89.74% of participants considered their training adequate to the post-graduate work requests, 10.26% identified areas that would require further training. There were no differences in satisfaction rates between classes (Table 2). Only one respondent (2.75% of the total) reported having faced professional challenges that were above their training level. The mean time-to-first-job was under 90 days and specifically 60.28 ±79.73 (sd), CI (34.43–86.12) with a *p*-value of 0.025 and there were no differences in terms of time-to-first-job based on the cohorts (Figure 1).

### 3.2. Secondary Endpoint

**Figure 2:**
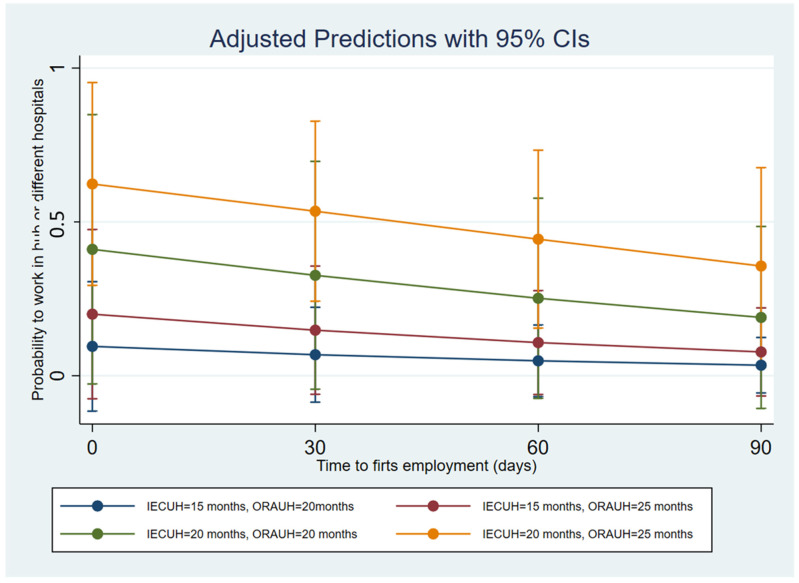
Shows how increasing the IECUH followed by a further ORAUH allows for more probability to work in hub hospitals; the graph describes an example of 4 scenarios with different periods of IECH and ORAUH and the probability to work in hub hospitals in the postgraduation period.

In the studied cohort, the primary training activity was ORAUH, spanning over an average of 30.41 ± 6.6 (sd), CI (28.47–32.42) months followed by IECUH with an average of 17.29 ± 4.49 (sd), CI (15.79–18.66) months. Training at one of the network facilities amounted to 8.08 ± 4.27 (sd), CI (6.88–9.03) months, while training abroad to 2.39 ± 3.39 (sd), CI (1.48–3.38) months. No differences were detected among school cohorts in medical training (Table 3). The multiple variables logistic regression showed a correlation among working in hub hospitals and IECUH, with an odds ratio of 1.46 CI (1.09–1.976) and *p*-value of 0.015 and ORAUH 1.18 CI (1.00–1.40) with a *p*-value of 0.047. The time-to-first-employment was not significative of an odds ratio of 0.98 (0.97–1.05) with *p*-value 0.184, ROC of the model 0.785 with *p*-value 0.07 (Figure 2 and Table 4).

## 4. Discussion

**Figure 3:**
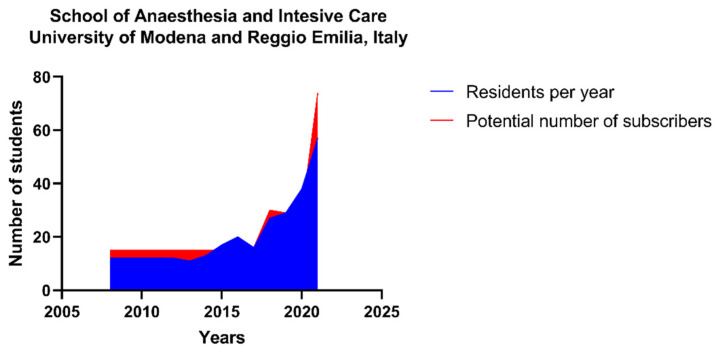
Trend of number of residences and potentials subscribers per year for the school of Anesthesia and Intensive Care of the University of Modena and Reggio Emilia (Italy).

Fajcikova et al. recently described the motivation of students to choose a specific university [8]. Al-Abri et al. analyzed the reasons to choose a particular career path [9]. Despite what is found in health literature studies: postgraduation schools are not typical, especially during the postgraduation period. How and which type of hospitals are chosen by new specialists in A-IC is not known. These results describe which curricula are preferred and which types of hospitals are mostly coveted. School cohorts have shown a similar choice: the predominant choice as first employment was in public hospitals as a hub and spoke. It could be explained that hospitals with several colleagues could help in case of needs. Despite over 85% of participants declaring to be satisfied with their medical training, working in hospitals with a high volume of procedures and team working could make them feel safer. Furthermore, public hospitals usually have more disciplines of A-IC, offering more possibilities to implement education and medical practice. School cohorts demonstrated similar curricula: attending more medical training in university hospitals can favor future employment in hub hospitals. The result can originate from previous partnerships and relationships of trust, or it could be the desire to confront stimulating scenarios with different disciplines. Moreover, university hospitals offer to be involved in research programs. Therefore, these results suggested that more months spent in medical training in university hospitals is propaedeutic to hub hospitals. The study described a time of stable employment, or the time needed to start working as a freelance significative lesser than 90 days. Considering the bureaucratic requirements or the time it takes to be hired in public hospitals, the time above described was quick. Obviously, the time to be hired is faster, but this fact is due to the pandemic event and the accompanying medical turnover, where the high demand for intensive care physicians has changed the training program, making some steps faster and linked to the complete autonomy [1,5,10,11]. These last concomitant event has generated a fast hospital turnover rate and it has not allowed for the conducting of a new survey recently; moreover, medical trainings and paths, due to the contingency of the moment, are different. Therefore, a comparison it should be difficult. The high demand of specialist in this field depends on that the A-IC filed has been growing in recent years. The non-operating room anesthesia field involves many procedures requiring the presence of an anesthesiologist for deep sedation for endoscopic gastroenterology, operative radiology, dental surgery, operative cardiology, and cath lab. The development of a large training network was instrumental in addressing this issue and increasing the training capacity of the school that has been growing progressively over the last ten years to 57 new residents per year in 2021 (Figure 3).

The school of A-IC of the UNIMORE offers to all students attending operating room and intensive care a one-to-one resident-consultant ratio: a model that facilitates the professional growth of the resident from close collaboration with the consultant to complete autonomy [12]. The A-IC school organization provides multiple rotations in several hospitals from the first years allowing all students to develop an adequate know-how and experience diversified professional approaches. Despite the central core of the training is always the university hospitals. The main activities present in most hospitals, such as general surgery, orthopedic surgery, obstetrician surgery, and intensive care, are repeated at least twice during the training, usually during the first two years, then with a different level of autonomy and awareness during the last to prepare the future specialist to the most likely professional challenges, as reported by post-graduate anesthesiologists. All training activities can be recorded online on a log-book, which records the type and number of activities and the level of autonomy [13,14]. The log-book can be printed as a record of the activities performed (Figure 2). The mission of the A-IC school is to offer all residents training on a common core of competence in line with the recommendations of the European and Italian Society of Anesthesia and Intensive Care [15,16,17]. The A-IC school of the UNIMORE network allowed students to attend several foreign centers with a specific target, such as Cardiocentro Ticino Institute of Lugano (CH) for cardiac surgery practice or Ospedale Pediatrico Bambino Gesù of Rome (SCV) for pediatric A-IC: the objective is to complete the local offer with external experiences to ensure the core curriculum required by MIUR is covered adequately. Furthermore, residents can attend several national and international meetings and are involved in variety research projects. In addition, inside the school network, there are also two high-fidelity simulation labs where is possible to implement technical and non-technical skills, has it happened for other disciplines [3,18,19,20,21,22]. Thanks to all these resources, the school of Modena is accredited by MIUR and currently trains 147 residents, and it has played a strategic role, as reservoir of physicians with a degree of training in A-IC of UNIMORE during many stressful system events such as Emilia-Romagna region (Italy) earthquake in 2012, and during the Vasco Rossi Modena Park^®^ concert with about 220.000 participants and about 5000 staff members [7]. During the COVID-19 pandemic, the A-IC school of UNIMORE, similar to others post graduated schools offered its support to the local and regional healthcare response and residents were also involved in the humanization process during a particular setting, such as hospitalization in COVID-19 intensive care [23,24].

### Strength and Limitations

**Figure 4:**
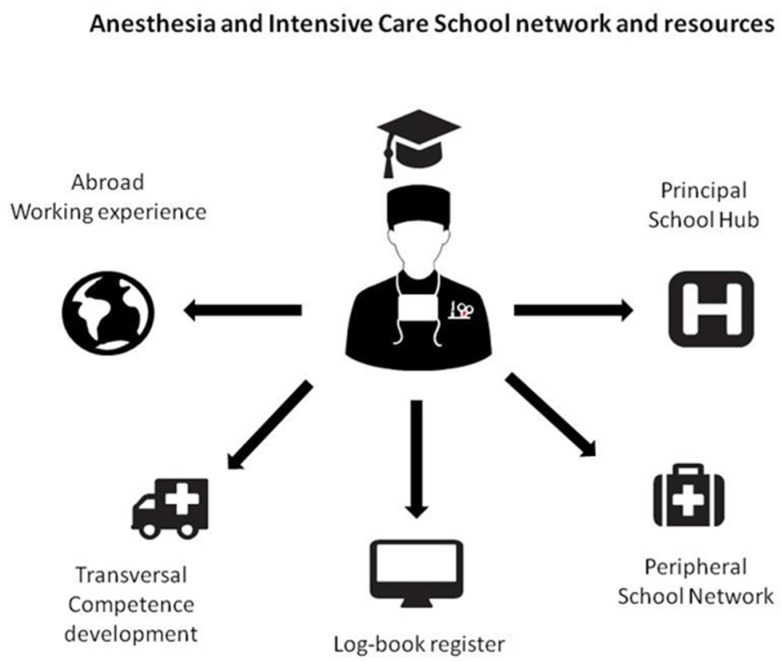
Anesthesia and Intensive Care school network and resources.

This study was performed at the end of 2018 and ended in 2019; due to pandemic events it was not possible collected data further data and performing a new study collecting data also about cohorts 2013–2018 and 2014–2019. Many new specialists were involved in the pandemic. The research describes the postgraduation period in the absence of pandemic events. This survey is maybe the first or one of the first studies in its field. This study has no conflict of interest on school of A-IC of UNIMORE. The study describes some exciting correlations between medical training and the type of hospital to determine a possible factor for a prospective trial. It was not easy to provide student population background for privacy reasons. Furthermore, it is difficult to make comparison results of other universities since this research is one of the first studies on this topic (Figure 4).

## 5. Conclusions

This paper describes the postgraduation period in A-IC. This last point should be examined as an integral part of the accreditation procedure. Knowing the satisfaction rate, perception autonomy, and which type of hospitals are preferred can measure the education training capacity of a postgraduation school. Furthermore, blind judgment feedback later in the first year of work could be an essential further measurement. These data suggested how university hospitals represent the central core of education. Currently the transition from postgraduation school to the clinical practice occurs seamlessly.

There is a growing demand for A-IC specialist filed by the recent pandemic and the expansion of non-operating room anesthesia procedures, that calls for an expansion of the training capabilities and capacity of the school.

The network must, therefore, meet the educational/cultural and practical needs necessary to adequately train specialists to meet the current challenges, ensuring the safety of the professional and the patient. Feedback from alumni can prove useful in assessing the performance of a postgraduation school.

## Figures and Tables

**Table 1:** Type of hospitals as first employment.

Type of Hospital after 12 Months	I 2009–2014	II 2010–2015	III 2011–2016	IV 2012–2017	
Spoke hospital	44.0%	27.7%	55.6%	11%	
Hub Hospital	22.2%	63.7%	33.3%	66.67%	
Private Hospital	33.0%	9.09%	11.1%	22.2%	
					0.248

**Table 2:** Post-graduated satisfaction test results.

	I 2009–2014	II 2010–2015	III 2011–2016	IV 2012–2017	*p* Value
Post graduated difficulties were present? (Yes)	11%	0%	0%	0%	0.317
Were all programs available to residents (No)	25%	18.8%	0%	0%	0.177
Percentage of Satisfaction (Yes)	88.89%	100%	90.91%	88.89%	0.736

**Table 3:** Training among school cohorts.

Months/Observations (obs)	I 2009–2014 (9 obs)	II 2010–2015 (11 obs)	III 2011–2016 (11 obs)	IV 2012–2017 (9 obs)	*p*-Value
ORAUH (mean ± std. dev)	29.4 ± 6.62	33.54 ± 5.78	27.81 ± 7.65	32.44 ± 5.70	0.188
IECUH (mean ± std. dev)	18.22 ± 2.68	15.45 ± 2.46	15.18 ± 5.87	18.44 ± 5.44	0.103
School Network months (mean ± std. dev)	7.55 ± 5.54	7.364 ± 4.22	9.18 ± 5.15	7.70 ± 3.86	0.769
Abroad Experience months (mean ± std. dev)	3.70 ± 3.84	2.54 ± 3.01	1.90 ± 2.34	1.33 ± 6.64	0.429

**Table 4:** Multiple variables logistic regression analysis.

Hub or Not	Odds.	St.Err.	t-Value	*p*-Value	[95% Conf	Interval]	Sig
IECUH	1.459	0.226	2.44	0.015	1.077	1.977	**
ORAUH	1.189	0.103	1.99	0.047	1.002	1.409	**
Time	0.988	0.009	−1.33	0.184	0.97	1.006	
Constant	0	0	−2.37	0.018	0	0.139	**
Mean dependent var		0.450		SD Dependent var		0.504	
Pseudo r-squared		0.216		Number of obs		40	
Chi-square		11.872		Prob > chi2		0.008	
Akaike crit. (AIC)		51.179		Bayesian crit. (BIC)		57.934	

** *p* < *0*.05.

## Data Availability

Not applicable.

## Appendix A

**Table A1:** School of Anesthesia and Intensive Care network of University of Modena and Reggio Emilia.

Health Facility	Site	Disciplines	Type of Hospital
Policlinico Teaching Hospital	Modena, Italy	Anesthesia and Intensive Care	Main domain of school: Poly-specialist hospital and pediatric and maternal hospital
Sant’Agostino Estense Public Hospital	Baggiovara (Modena), Italy	Anesthesia and Intensive Care	Main domain of school: Trauma Center and neuro critical care
Bernardo Ramazzini Public Hospital	Carpi (Modena), Italy	Anesthesia and Intensive Care	Peripheral Spoke
Santa Maria Nuova Public Hospital	Reggio Emilia, Italy	Anesthesia and Intensive Care	Principal hospital of Reggio Emilia, all disciplines excluded cardiac surgery
Public Hospital	Guastalla (Reggio Emilia), Italy	Anesthesia and Intensive Care	Peripheral Spoke

**Table A2:** School of Anesthesia and Intensive Care network of University of Modena and Reggio Emilia.

Health Facility	Site	Disciplines	Type of Hospital
Regina Margherita Public Hospital	Castelfranco Emilia (Modena), Italy	Chronic Pain Service	Chronic Pain Service
Villa Salus Private Hospital	Reggio Emilia, Italy	Anesthesia and Intensive Care	Cardiac surgery Center
Bellaria Public Hospital	Bologna, Italy	Anesthesia and Intensive Care	Neurocritical care Center
Montecatone Rheabilitation Center Private hospital	Imola (Bologna), Italy	Post critcal care Rehabilitation	Post critical care rehabilitation
118 Service	Modena, Italy	Pre-hospital emergency care	Pre-hospital emergency care

**Table A3:** Program of school of Anesthesia and Intensive Care of University of Modena and Reggio Emilia.

School Program	Field	Mandatory or Optional	Year
General surgery	Anesthesia-Operating room	Mandatory	I-II and IV-V year
Orthopedic surgery	Anesthesia-Operating room	Mandatory	I-II and IV-V year
Obstetrician surgery	Anesthesia-Operating room	Mandatory	I-II and IV-V year
Urology surgery	Anesthesia-Operating room	Mandatory	I-II year
Otolaryngology surgery	Anesthesia-Operating room	Mandatory	I-II and IV-V year
Oral Maxillo-surgery	Anesthesia-Operating room	Mandatory	I-II year
Plastic Surgery	Anesthesia-Operating room	Mandatory	I-II year
Endoscopic Gastroenterology	Anesthesia-Operating room	Mandatory	II-IV year
Operative Radiology	Anesthesia-Operating room	Mandatory	II-IV year
Post-operative Intensive Care	Intensive Care and Resuscitation	Mandatory	II and IV-V year

**Table A4:** Program of school of Anesthesia and Intensive Care of University of Modena and Reggio Emilia.

School Program	Field	Mandatory or Optional	Year
Echo-cardio Lab	Transversal competence	Optional	III-IV year
Radiology Unit	Transversal competence	Optional	III-IV year
Nephrology Unit	Transversal competence	Optional	III-IV year
Pediatric Surgery	Anesthesia-Operating room	Mandatory	III-IV year
Vascular surgery	Anesthesia-Operating room	Mandatory	III-IV year
Neurosurgery	Anesthesia-Operating room	Mandatory	III-IV year
Cardiac Surgery	Anesthesia-Operating room	Optional	IV year
Transplant Surgery	Anesthesia-Operating room	Optional	IV year
Neurocritical care	Intensive Care and Resuscitation	Mandatory	IV year
Pre-hospital emergency	Intensive Care and Resuscitation	Mandatory	IV year
Chronic Pain Management	Pain Management	Mandatory	IV year
Hyperbaric Therapy	Hyperbaric Therapy	Mandatory	IV-V year
Abroad Experience	Transversal competence	Optional	IV-V year
